# Neural mechanisms of different types of envy: a meta-analysis of activation likelihood estimation methods for brain imaging

**DOI:** 10.3389/fpsyg.2024.1335548

**Published:** 2024-03-19

**Authors:** Shuchang Dai, Qing Liu, Hao Chai, Wenjuan Zhang

**Affiliations:** ^1^College of Education and Technology, Zhejiang University of Technology, Hangzhou, China; ^2^Mental Health Education Center, Xidian University, Xi’an, Shaanxi, China

**Keywords:** envy, functional magnetic resonance imaging, activation likelihood estimation, meta analyses, neural mechanism

## Abstract

Previous studies have a lack of meta-analytic studies comparing the trait (personality) envy, social comparison envy, and love–envy, and the understanding of the similarities and differences in the neural mechanisms behind them is relatively unclear. A meta-analysis of activation likelihood estimates was conducted using 13 functional magnetic resonance imaging studies. Studies first used single meta-analyses to identify brain activation areas for the three envy types. Further, joint and comparative analyses were followed to assess the common and unique neural activities among the three envy types. A single meta-analysis showed that the critical brain regions activated by trait (personality) envy included the inferior frontal gyrus, cingulate gyrus, middle frontal gyrus, lentiform nucleus and so on. The critical brain regions activated by social comparison envy included the middle frontal gyrus, inferior frontal gyrus, medial frontal gyrus, precuneus and so on. The critical brain regions activated by love–envy included the inferior frontal gyrus, superior frontal gyrus, cingulate gyrus, insula and so on. In terms of the mechanisms that generate the three types of envy, each of them is unique when it comes to the perception of stimuli in a context; in terms of the emotion regulation mechanisms of envy, the three types of envy share very similar neural mechanisms. Both their generation and regulation mechanisms are largely consistent with the cognitive control model of emotion regulation. The results of the joint analysis showed that the brain areas co-activated by trait (personality) envy and social comparison envy were frontal sub-Gyral, inferior parietal lobule, inferior frontal gyrus, precuneus and so on; the brain areas co-activated by trait (personality) envy and love–envy were extra-nuclear lobule, lentiform nucleus, paracentral lobule, cingulate gyrus and so on; the brain regions that are co-activated by social comparison envy and love–envy are anterior cingulate gyrus, insula, supramarginal gyrus, inferior frontal gyrus and so on. The results of the comparative analysis showed no activation clusters in the comparisons of the three types of envy.

## 1 Introduction

Envy is a psychological and behavioral activity prevalent in human societies. For individuals, it is a rich emotional experience. Envy is considered as a combination of the primary emotions anger, fear and sadness (Zheng et al., 2019). Along with the experience of an unpleasant emotional state, envy also is associated with a host of behaviors. Extreme, pathological envy includes delusional symptoms and promotes aggression in terms of domestic violence, self-mutilation and even murder (Camicioli, 2011) and can also occur in association with depression and autism (Bauminger, 2004). Conversely, positive outcomes related to envy also have been reported, including motivating people to do better than their competitor (Protasi, 2016), by for example, inspiring individuals to improve their position in the workplace. For groups, envy is also a complex social culture and phenomenon (Pang, 2016). Envy usually occurs in social interactive contexts, such as sexual infidelity or social comparison scenarios. From an evolutionary perspective, envy prevents an individual from being outperformed by a direct competitor in a fitness-relevant domain: Envy motivates behaviors towards gaining a similar standing as a competitor or acting to remove a competitor’s advantage. Therefore, We experience envy when the positive attributes of another individual jeopardize our social standing (Crusius and Lange, 2016).

The phenomenon of envy is complex, and to understand it more clearly, based on scientific research, psychologists have categorized envy. According to Bringle’s (1991) categorization, envy can be broadly classified into two types - suspicious envy and reactive envy. Bringle’s Interaction Model Theory of Envy states that envy reactions are the outcome of an interaction between endogenous (internal) and exogenous (external) variables, such as the environment and culture. However, the impact of these variables may differ in each individual, leading to different types of envy. When the internal variable plays a significant role in determining the envy response, it is known as suspicious envy. On the other hand, if the external variable is an important determinant, it is called reactive envy. Currently, the three types of envy commonly accepted by the general public and the subject of much research are the trait (personality) envy, social comparison envy, and love–envy. Of the three types of envy, suspicious envy is typified by trait (personality) envy. Outcomes from a relationship, comparison level (CL), and comparison level of alternatives (CLalt) are typically viewed as situational determinants of envy (Bringle, 1981). The Bringle Self-report Jealousy Scale (BSJS), which was developed by Bringle et al. (1979), is a tool that measures an individual’s experience of self-envy in different contexts. It does so by using two dimensions - Social Comparison Envy and Love–Envy. It can be seen that reactive envy is typified by social comparison envy and love–envy. They all have their own unique characteristics.

The response of trait (personality) envy is mainly determined by endogenous variables, which are related to individuals. Envy is thought to arise from the perceived threat of losing respect and social status in the eyes of others (Silver and Sabini, 1978; Fiske, 2010; Crusius and Lange, 2016). Unlike situational envy, which manifests based on specific tasks, trait (personality) envy exhibits a general sensitivity to status threats. However, the tendency to react negatively emotionally and the corresponding behavioral changes vary across individuals. Empirical research has shown that individuals differ in the extent to which they desire social status (Anderson et al., 2015) and compare themselves to others (Gibbons and Buunk, 1999), and thus differ in their tendency to experience envy when faced with upward social comparison. Moreover, comparison-related personality disposition traits (Gibbons and Buunk, 1999) may shape trait (personality) envy, such as inequity aversion, justice sensitivity, and achievement motivation (Steinbeis and Singer, 2013; Lange and Crusius, 2015a). As for the measurement of the trait (personality) envy, researchers have independently developed and validated various scales, including the Envy subscale of the Materialism Scale developed by Belk (1985), the Multidimensional Jealousy Scale (MJS) developed by Pfeiffer and Wong (1989), the York Enviousness Scale (YES) developed by Gold (1996) from York University, Canada, the Dispositional Envy Scale (DES) developed by Smith et al. (1999), a nine-point envy scale developed by Lange and Crusius (2015b).

The response of social comparison envy and love–envy is mainly determined by exogenous variables, which are related to social and cultural. According to the theory of social comparison envy, social comparison is an essential aspect of social interaction. This process involves individuals comparing their beliefs, attitudes, and opinions with those of others (Festinger, 1954). However, when individuals engage in unfavorable upward social comparisons, they may experience painful feelings of envy (Silver and Sabini, 1978; Salovey and Rodin, 1984). Furthermore, behavioral research on envy has also confirmed that the more a person compares themselves to others, the more jealous experiences they will experience (Smith et al., 1999; Zeelenberg and Pieters, 2007). Love–envy is considered to be an emotion experienced when an individual faces the loss of an existing significant relationship with another person (meaning a companion) because of a third person (Mathes, 1992), which includes love–envy resulting from infidelity (sexual or emotional infidelity). Love–envy can sometimes have adverse effects, particularly triggering behaviors such as excessive snooping, controlling companions, and verbal or physical aggression (Kar and O’Leary, 2013; Neal and Lemay, 2014). These behaviors can damage intimate relationships between companions and may even lead to malignant events, such as domestic violence (Dandurand and Lafontaine, 2014; Deans and Bhogal, 2019). Social comparison envy and love–envy were the two most common types of reactive envy. The Bringle Self-Report Envy Scale (BSJS), developed by Bringle et al. (1979), includes both social comparison envy and love–envy dimensions to measure individuals’ extensive experiences of self-envy in various contexts. In contrast, the Interpersonal Relationship Scale (IRS) developed by Hupka and Bachelor (1979) measured a single envy type.

Many persons view envy as neutral: It is neither only good nor only bad. Thus, elimination of all envy is not necessarily a desirable outcome. One should be prepared to cope with real, impending threats. Managing envy so that it becomes a constructive factor in a relationship is desirable. Thus, one should explore positive ways to cope with feelings. Self-management of envy is closely linked to emotion regulation. Emotion regulation includes a wide range of cognitive, behavioral, emotional, and physiological responses and is necessary to understand the emotional and behavioral correlates of stress and negative emotional states (Garnefski and Kraaij, 2006). Ochsner and Gross (2007) constructed a cognitive control model of emotion regulation with a bottom-up and top-down perspective. According to the theory, generating emotions involves four stages. In the first stage, a stimulus is perceived in its current situational context. At the second stage, one attends to some of these stimuli or their attributes. The third stage involves appraising the significance of stimuli in terms of their relevance to one’s current goals, wants or needs. Finally, the fourth stage involves translating these appraisals into changes in experience, emotion-expressive behavior, and autonomic physiology (Ochsner and Gross, 2008). Cognitive reappraisal and expressive inhibition are two strategies for regulating emotions. The cognitive control model of emotion regulation suggests that emotion regulation arises during the process of emotion onset and that different emotion regulation occurs at different stages of emotion onset (Gross and James, 1998; Gross and Thompson, 2007). Among them, cognitive changes are formed before the formation of emotional response tendencies, which are prior-focused emotion regulation and exhibit cognitive reappraisal emotion regulation strategies; response adjustments are made after the formation of emotional response tendencies, which are response-focused emotion regulation and exhibit expression inhibition of emotion regulation strategies.

Previous studies have addressed the neural mechanisms underlying envy less frequently, and only a very few studies have examined the neural mechanisms associated with non-pathological envy in healthy individuals. These researchers have used brain imaging techniques such as functional magnetic resonance imaging (fMRI) and other brain imaging techniques to explore the neural mechanisms underlying different types of envy, trying to find structural and functional markers associated with envy in the brain. In an fMRI study on trait (personality) envy, Xiang et al. (2016) used regional homogeneity (ReHo) to measure trait (personality) envy. They found that the inferior frontal gyrus (IFG), middle frontal gyrus (MFG), and dorsomedial prefrontal cortex (DMPFC) were found to be positive predictors of personality envy; Xiang et al. (2017) used a voxel-based morphometry (VBM) approach to measure trait (personality) envy and found that trait (personality) envy was positively correlated with dorsolateral prefrontal cortex (DLPFC) and superior temporal gyrus (STG) were positively correlated; Zheng et al. (2019) used a neural representation of emotions to measure trait (personality) envy and found that insula, fusiform gyrus (FG), hippocampus, dorsal striatum (DS), and inferior frontal gyrus (IFG) were found to have increased activation in these brain regions. In an fMRI study on social comparison envy, Dvash et al. (2010) found activation of the ventral striatum (*VS*) by inducing social comparison envy through a money gain or loss game; Tanaka et al. (2019) used a slightly different money gain or loss game than the former paradigm to induce social comparison envy and found that the dorsal anterior cingulate cortex (dACC) was activated; Brennan et al. (2020) used a story context approach to induce social comparison envy and found that the superior frontal gyrus (SFG) was significantly activated with increasing levels of envy. In fMRI studies on love–envy, Katrin et al. (2015) used an infidelity contextual utterance task to induce love–envy and found that the anterior cingulate cortex (ACC) was activated, while Sun et al. (2016) used a contextual imagery task to induce love–envy and found that the ventral medial prefrontal cortex (VMPFC) was activated.

Previous studies have some limitations. For instance, empirical research has its own inherent shortcomings. Firstly, individual brain imaging studies tend to involve a relatively small number of subjects. This may lead to low statistical test power and effect sizes (Yarkoni, 2009). Secondly, neuroimaging results may be inconsistent due to the sensitivity of the task and control conditions selected. Thirdly, Single fMRI studies often focus only on specific activated brain regions related to envy, disregarding the broader mechanisms responsible for generating and regulating it. Therefore, meta-analysis techniques based on large-scale data synthesis methods are necessary to overcome the limitations of individual brain imaging studies (Yarkoni et al., 2011). This method not only helps to make up for the lack of understanding of the three envy types as a whole, but also explores the generality and variability of neural activity among the three types, and provides representative reference coordinate points for future region of interest (ROI) analyses. However, there is a lack of meta-analytic studies comparing the trait (personality) envy, social comparison envy, and love–envy, and the understanding of the similarities and differences in the neural mechanisms behind them is relatively unclear.

Activation likelihood estimation (ALE) meta-analysis is an unbiased and objective approach to analyzing brain function (Wager et al., 2007). It can provide a consistent quantitative measure of relevant studies in this research area. Notably, the ALE meta-analysis method effectively avoids the problems of low statistical test power and high false-favorable rates in individual neuroimaging studies (Button et al., 2013; Eklund et al., 2016). Therefore, this study analyzed the existing functional magnetic resonance imaging (fMRI) studies of three envy types, namely trait (personality) envy, social comparison envy, and love–envy, by ALE and observed the similarities and differences in the processing brain regions of the three envy types to identify the neural mechanisms underlying the processing of the three envy types.

## 2 Methods

### 2.1 Literature search and inclusion criteria

This study used the CNKI full-text database to search for Chinese literature and PubMed, Web of Science, Elsevier Science Direct, Semantic Scholar, and ProQuest databases to search for foreign language literature. To conduct the literature search, we used the keywords “envy” and “fMRI” for Chinese sources and “envy,” “fMRI,” or “envy and fMRI” (adapted for the Web of Science database format) for foreign sources. After the screening, we obtained 425 papers. Further, after reading the abstract, methods, and results sections of each article, those that met the following six characteristics were included in the meta-analysis:

1. The type of study in question is empirical literature, which excludes reviews, meta-analyses, and case studies.
2. The research content excludes experienced envy, attributed envy, benign envy, malicious envy, and other types of envy less studied, focusing on trait (personality) envy, social comparison envy, and love envy.
3. The study only included normal individuals as subjects. Patients with brain lesions, neurological conditions, juvenile delinquents, and other special groups whose brain structure and function have been significantly altered were not part of the meta-analysis.
4. The research methodology involved subjects completing a scale or an experimental task related to jealousy, with an experimental comparison condition related to jealousy (contrast). The study used the fMRI method, excluding other methods like electroencephalography (EEG), magnetoencephalography (MEG), diffusion tensor imaging (DTI), which were used to analyze the condition of white and gray matter, and magnetic resonance imaging (MRI).
5. Whole-brain analyses were used, excluding studies with only region of interest analysis.
6. The study provided the coordinates of the brain regions that were found to be activated during the experiment. The activation results were reported using the standardized Talairach or MNI space. We excluded studies that did not report the coordinates of the activated regions. We also removed peak coordinates that were unrelated to envy and only activated by the evoked task, such as the peak activation coordinates in the visual cortex.

**Figure 1:**
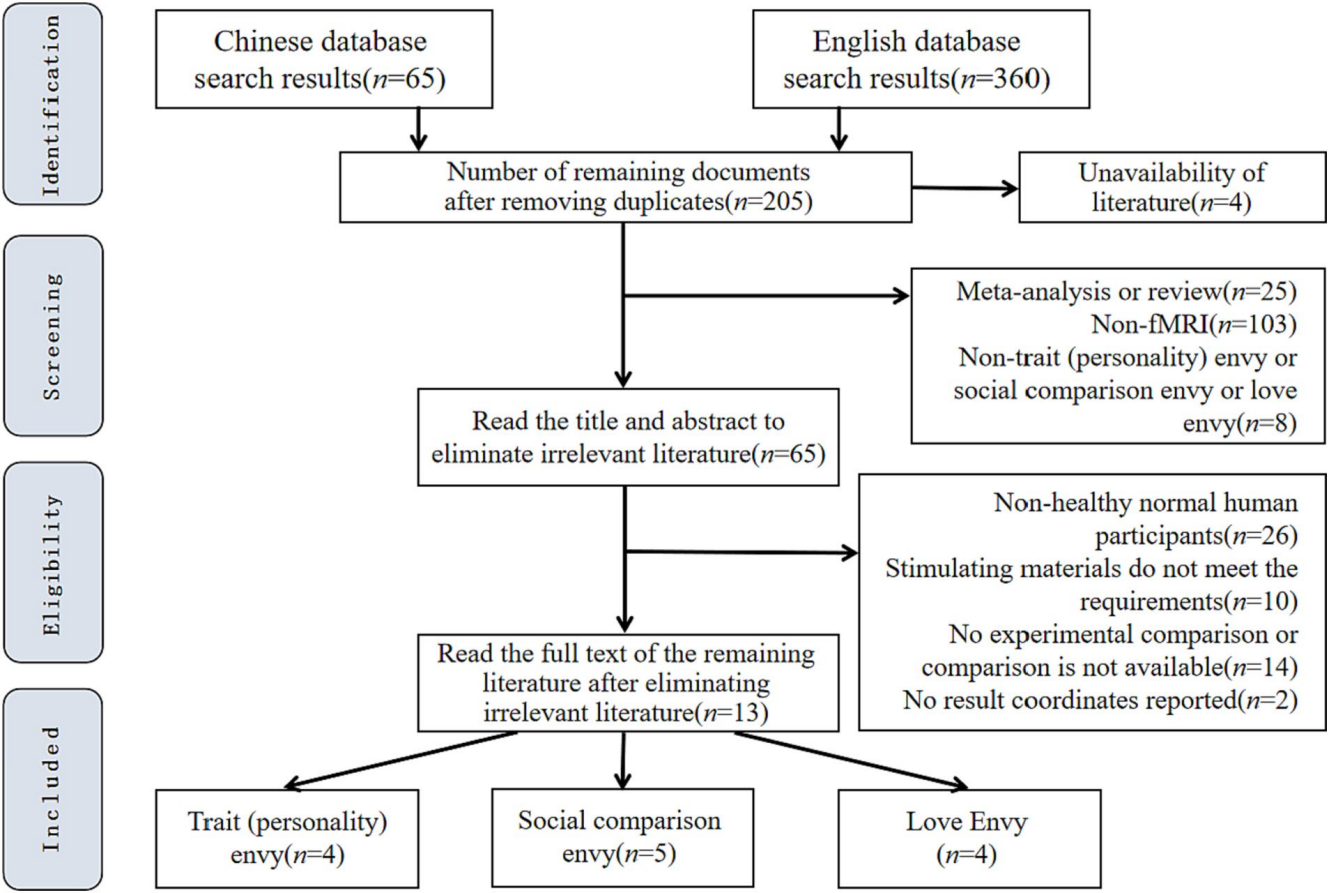
Flow chart of literature screening.

A total of 13 papers finally met the above criteria and were included in the present study’s meta-analysis. Figure 1 shows the specific screening process and results.

### 2.2 Systematic review

We followed recent recommendations on how to conduct a proper neuroimaging meta-analysis (Müller et al., 2018). For the current meta-analysis, 13 studies met the inclusion criteria reported in the previous section. Table 1 provides the basic information on the included studies.

**Table 1:** Details of the 13 included literature.

Types of envy	Literature information	*N*, Sex	Age	Contrasts	Peak	Coordinate system
Trait (personality) envy	Zheng (2021)	218, 107F	21.42	2, Angry, baseline	20	MNI
	Zheng et al. (2019)	92, 45F	21,68	5, Angry, happy, fear, sad, neutral	21	MNI
	Xiang et al. (2017)	27, 16F	20.63	2, Dispositional envy, neutral	2	MNI
	Xiang et al. (2016)	41, 24F	21,37	2, Dispositional envy, neutral	2	MNI
Social comparison envy	Sol et al. (2023)	58, 27F	27.86	2, Envy, neutral	15	MNI
	Daniel et al. (2021)	39, 0F	17.16	3, Positive, negative, neutral outcomes	11	MNI
	Brennan et al. (2020)	19, 10F	27.2	3, SpHi (superior with high similarity) condition, SpLo (superior with low similarity) condition, AvLo (average with low similarity) condition	4	MNI
	Tanaka et al. (2019)	97, 41F	19.3	2, Ro (reward for other)、Rs (reward for self)	1	MNI
	Dvash et al. (2010)	18, 10F	26.76	5, The absolute gain events, the other’s greater gain, the absolute loss events, the other’s greater loss, no change	13	Talairach
love–envy	Nadine et al. (2019)	11, 11F	29.9	3, Jealousy Condition (JC), Control Condition (CC), Nonsense words (NC)	62	MNI
	Sun et al. (2016)	37, 18F	22.8	2, Happiness scenarios, Jealousy scenarios	15	MNI
	Katrin et al. (2015)	22, 0F	26.73	3, Sexual infidelity, Emotional infidelity, Neutral	23	MNI
	Takahashi et al. (2006)	19, 9F	22.1	6, Men (Sexual infidelity, Emotional infidelity, Neutral), Women (Sexual infidelity, Emotional infidelity, Neutral)	21	MNI

The MNI space is a coordinate system created by the Montreal Neurological Institute based on a series of magnetic resonance images of the average human brain. The Talairach coordinate system is based on the standard brain anatomy atlas established by French anatomist Talairach.

Data were extracted from the studies and then checked. We then created a database containing the following information of the selected articles: type of envy, literature information (author and publication date), number of participants and among them the number of female participants, average age of participants, experimental comparison conditions, number of activation peaks reported for the experiments, and coordinate space (Talairach or MNI space).

The 13 included literature reported three types of envy, a total of 698 participants. In the literature on trait (personality) envy, there were 186 male participants and 192 female participants. In the literature on social comparison envy, there were 143 male participants and 88 female participants. In the literature on love–envy, there were 51 male participants and 38 female participants. The included literature had an average age ranging from 17 to 27 years. Their average age ranged from 17 to 27 years. The 13 included literature also reported 40 experimental comparison conditions and 216 peaks. For most functional neuroimaging meta-analyses, it is important to explicitly incorporate the paradigm of the literature (Müller et al., 2018). This paper considered all paradigms for different types of envy and focused on the higher order supervisory control processes necessary in all paradigm types. Of the 40 comparison conditions, trait (personality) envy included three approaches: regional homogeneity (ReHo), voxel-based morphometry (VBM), and neural representations of emotion. There are two main task types to induce social comparison envy - the story context method and the money gain/loss game; the story context method causes the SpHi condition (SpHi = superior with high similarity), the SpLo condition (SpLo = superior with low similarity), AvLo condition (AvLo = average with low similarity) three scenarios or target character and positive or unfortunate (fortunate/neutral) events, the money gain/loss game induces gain, loss, no change or Ro (reward for other) and Rs (reward for self) game outcomes. The infidelity contextual statement task comprises sexual infidelity, emotional infidelity, and neutral contextual statements. The contextual imagination task involves imagining a familiar friend with the love rival “A,” the love object “B,” and the love rival “Jack.” It includes two scenarios: happiness scenarios, where the participant imagines themselves with “A” or “B,” envy scenarios, where the participant imagines “Jack” interacting with “A” or “B,” and “A” or “B” interaction scenarios.

### 2.3 Publication bias

Publication bias is a problem that should be addressed. That is, there is in general in science a bias to publish mainly significant results while experiments failing to reject the null-hypothesis are often not reported (Ioannidis et al., 2014). Publication bias seriously impacts the reliability of meta-analysis results and overestimates the existing average effect. Several methods are generally used to test for publication bias meta-analyses, including the funnel plot, Begg test, classic fail-safe *N* test, Egger’s test, and *p*-curve test. These tests can help determine whether there is significant publication bias in a meta-analysis. In this study, a funnel chart and Begg test were used to test for publication bias.

Note that the literature we included was all related to the study of various types of envy and fMRI. Since the data included in the meta-analysis were the fMRI coordinates used in various literature, publication bias could not be determined from this. Therefore, this paper uses the main effect size of each literature to judge the problem of publication bias.

### 2.4 Activation likelihood estimation method

ALE analysis is a meta-analytic technique that evaluates the co-localization of reported activations across studies. The first step is to categorize experiments in the literature, such as by stimulus or task. Whole-brain probability maps are then created across the reported foci in standardized stereotaxic space (Talairach or MNI). To create probability maps, this meta-analysis used GingerALE software. The probabilities are modeled by 3D Gaussian density distributions that adjust the FWHM for each study to account for sample size variability. For each voxel, GingerALE estimates the cumulative probabilities that at least one study reports activation for that locus. This generates a statistically thresholded ALE map, accounting for spatial uncertainty across reports. The resulting ALE values reflect the probability of reported activation at that locus, with high values indicating high probability estimates. This value is tested against the null hypothesis that activation is independently distributed across all studies in the meta-analysis, using random effects (Turkeltaub et al., 2012).

The GingerALE software (V3.0.2)^1^ was used to process the data. However, there was a problem of inconsistency in the peak coordinate system as some studies used the Talairach coordinate system while others used the MNI (Montreal Neurological Institute) coordinate system based on the standard brain template on which the peak coordinates were derived. To address this issue, before analyzing the data, the coordinate systems of the studies included in the analysis were converted using the Convert Foci tool in the GingerALE software. The “Brett: MNI to Talairach” option was selected to convert the reported coordinates from MNI space to Talairach space. This conversion is done automatically when the data are inserted into the BrianMap database using a transform called icbm2tal developed by Lancaster et al. (2007). This new transform provides improved fit over the Brett transform (mni2tal), and improves the accuracy of meta-analyses (Laird et al., 2010).

Afterward, the process of “Single Dataset” was carried out for the three types of envy. Talairach contrast coordinates of activation from eligible envy studies were combined (use the “Save and Merge Foci” tool in GingerALE) to create 3D maps depicting the likelihood of activation within each voxel in an fMRI template. Significant areas were identified depending on whether the envy processing location was more likely to occur in comparison to random spatial distributions. Analyses were thresholded using a cluster-lever FWE for multiple comparisons at *p* = 0.05. The FWE corrected threshold is set to the ALE value that no more than a specified fraction of the distribution exceeds that value. FWE thresholds are more conservative, so 5% of random studies, or *p* < 0.05 is recommended (Eickhoff et al., 2016). Finally, using multiple comparisons (5,000 alignments) correction at a clustering threshold of *p* < 0.001 (Liu et al., 2022).

Contrast analyses were performed to identify common (i.e., conjunction) and significantly different brain areas involved in trait (personality) envy, comparison envy, and love envy. Since the contrast analyses used ALE maps thresholded for multiple comparisons, the threshold was set to uncorrected *p* = 0.01 (10,000 permutations, 200 mm^2^ minimum volume for contrasts; Arsalidou et al., 2020). With these options, GingerALE software allows for between group comparisons, however, currently there are no options for correlational analyses.

Once the thresholded map has been created, we’ll need an anatomical underlay in order to view the meta-analysis results in context. Mango (Multi-Image Analysis GUI)^2^ is a viewer for biomedical research images developed by Jack Lancaster and Michael Martinez. We use the “Colin_tlrc_ 2 × 2 × 2. Nii (dimensions match GingerALE images)” to view our meta-analysis results on Mango (V4.1).

## 3 Results

### 3.1 Publication bias

**Figure 2:**
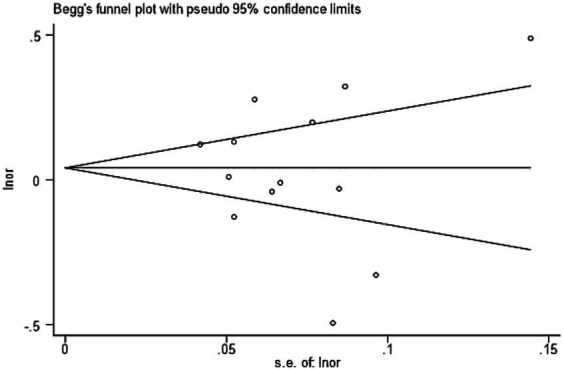
The result of funnel diagram.

Through the funnel diagram, it can be found that the effect distribution represented by 13 included literature is roughly symmetrical in the funnel plot, in which there are six points above the average effect value and seven points below the average effect value. Both above and below, there are only three points outside the 95% confidence region. Therefore, the funnel plot can show that there is no publication bias in the included literature research. The *p* value obtained by the Begg test is 0.760, which also confirms the above view. The result of funnel diagram is shown in Figure 2.

### 3.2 Single meta-analysis results

**Figure 3:**
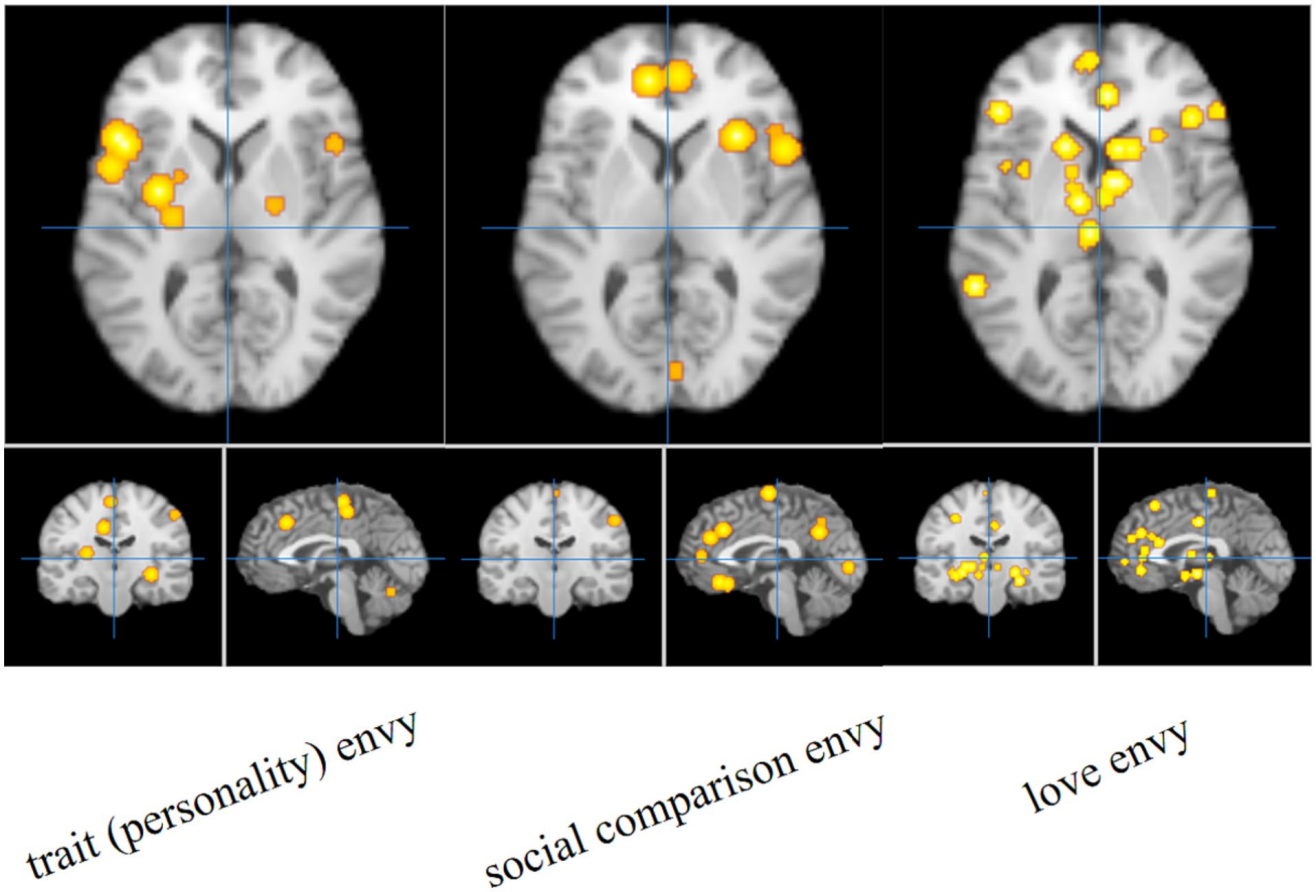
Single meta-analysis of the three envy types.

The single meta-analysis showed that 45 peak copulas for trait (personality) envy yielded 20 clusters, 44 peak copulas for social comparison envy yielded 25 clusters, and 121 peak copulas for love–envy yielded 45 clusters (Tables 2–4). Calculation of the peak distribution ratios for each region revealed that trait (personality) envy-related peaks were mainly distributed in the frontal lobe (35%), parietal lobe (15%), posterior lobe (12%), Sub-lobar (12%), limbic lobe (9%), temporal lobe (8%), occipital lobe (5%), and anterior lobe (4%); social comparison envy-related peaks were mainly distributed in the frontal lobe (48%), parietal lobe (15%), temporal lobe (9%), posterior lobe (8%), limbic lobe (8%), occipital lobe (6%), sub-lobar (4%), anterior lobe (2%); love–envy-related peaks were mainly distributed in the frontal lobe (30%), sub-lobar (22%), limbic lobe (17%), parietal lobe (12%), temporal lobe (9%), occipital lobe (4%), posterior lobe (3%), anterior lobe (3%). Specifically, trait (personality) envy was activated mainly in the inferior frontal gyrus, cingulate gyrus, middle frontal gyrus, lentiform nucleus, inferior parietal lobule, declive, and superior frontal gyrus; social comparison envy was activated mainly in the middle frontal gyrus, inferior frontal gyrus, medial frontal gyrus, precuneus, inferior parietal lobule, precentral gyrus, superior temporal gyrus, declive, and anterior cingulate gyrus; love–envy was activated mainly in the inferior frontal gyrus, superior frontal gyrus, cingulate gyrus, insula, claustrum, medial frontal gyrus, inferior parietal lobule, caudate, and posterior cingulate gyrus. The distribution of brain activation in the three envy types is shown in Figure 3.

**Table 2:** Single meta-analysis of trait (personality) envy.

Cluster#	Volume mm^3^	Hemisphere	Brain region	Peak coordinates (MNI coordinate system)			ALE Value (×10^−4^)
				X	Y	Z	
1	7,368	R	FL (IFG)	30	18	−14	9.52
		R	Sub-lobar	36	4	−8	9.52
		R	FL (IFG)	44	16	−6	9.51
		R	FL (IFG)	32	30	−4	9.44
2	7,224	L	LL (AC)	−12	24	26	9.54
		L	FL (CC)	−12	22	36	9.49
		R	LL (CC)	2	22	38	9.47
		L	FL (SFG)	−20	20	48	9.45
3	5,888	L	TL (SFG)	−50	4	2	9.52
		L	FL (Prec)	−44	14	6	9.50
		L	TL (MTG)	−54	2	−8	9.50
		L	FL (Prec)	−48	16	6	9.47
4	5,520	L	Sub-lobar (LN)	−24	−18	10	9.48
		L	Sub-lobar (LN)	−30	−6	4	9.47
		L	Sub-lobar (LN)	−20	0	12	9.45
5	5,360	R	PL (IPL)	44	−34	36	9.51
		R	PL (Prec)	54	−28	44	9.51
		R	PL (IPL)	44	−48	38	9.48
6	4,960	\	\	24	24	32	9.50
		R	FL (Sub-lobar)	20	22	40	9.48
		R	FL (MFG)	32	26	26	9.48
7	4,952	L	PL (Precuneus)	−24	−64	38	9.49
		L	PL (Precuneus)	−14	−56	54	9.49
		L	PL (Precuneus)	−20	−62	46	9.48
8	4,648	R	FL (MFG)	48	30	16	9.49
		R	FL (MFG)	50	30	22	9.49
		R	FL (MFG)	50	16	28	9.44
9	4,488	L	FL (IFG)	−44	18	−12	9.49
		L	FL (IFG)	−26	22	−10	9.49
		L	FL (IFG)	−38	18	−10	9.43
10	3,712	R	Sub-lobar (LN)	20	−12	0	9.48
		R	Sub-lobar (LN)	32	−18	−8	9.46
11	3,656	L	PL (IPL)	−54	−34	40	9.51
		L	PL (IPL)	−42	−42	38	9.44
12	3,328	L	FL (ParL)	−2	−30	48	9.45
		L	FL (ParL)	−2	−28	56	9.43
13	1872	R	PL	38	−64	−24	9.48
14	1856	L	LL (CG)	−8	−26	34	9.45
15	1848	L	FL (SFG)	−18	48	20	9.48
16	1840	L	PoL	−6	−70	−24	9.46
17	1840	L	PoL	−12	−58	−14	9.48
18	1832	R	TL (FG)	36	−42	−14	9.52
19	1824	R	OL (IOG)	44	−72	−6	9.46
20	1824	R	FL (IFG)	48	14	12	9.54

FL, frontal lobe; IFG, inferior frontal gyrus; LL, limbic lobe; AC, anterior cingulate; CG, cingulate gyrus; SFG, superior frontal gyrus; TL, temporal lobe; Prec., precentral gyrus; ParL, paracentral lobule; MTG, middle temporal gyrus; LN, lentiform nucleus; PL, parietal lobe; IPL, inferior parietal lobule; MFG, middle frontal gyrus; PoL, posterior lobe; FG, fusiform gyrus; OL, occipital lobe; IOG, inferior occipital gyrus.

**Table 3:** Single meta-analysis of social comparison envy.

Cluster#	Volume mm^3^	Hemisphere	Brain region	Peak coordinates (MNI coordinate system)			ALE Value (×10^−4^)
				X	Y	Z	
1	9,464	R	PL (Precuneus)	12	−64	48	9.54
		R	PL (Precuneus)	12	−56	44	9.51
		L	PL (Precuneus)	−2	−58	30	9.50
		R	PL (Precuneus)	8	−50	40	9.49
		R	PL (Precuneus)	8	−42	46	9.46
		R	PL (Precuneus)	6	−62	38	9.45
2	7,656	L	FL (SFG)	−18	42	26	9.55
		R	LL (CG)	2	25	32	9.49
		L	FL (MFG)	−30	34	34	9.48
		L	LL (AG)	−2	36	24	9.42
3	5,792	L	TL (STG)	−38	8	−30	9.49
		L	LL	−28	6	−20	9.48
		L	LL (PG)	−30	−6	−14	9.47
4	5,176	R	FL (Prec)	52	12	8	9.51
		R	FL (IFG)	52	6	14	9.50
		R	FL (IFG)	48	20	0	9.46
5	3,824	R	PL (PoG)	54	−28	40	9.47
		R	PL (SG)	56	−36	32	9.45
6	3,720	L	LL (AC)	−8	42	8	9.50
		R	LL (AC)	5	44	10	9.43
7	3,704	R	FL (MFG)	38	40	22	9.55
		R	FL (MFG)	30	34	28	9.53
8	3,440	R	FL (MeFG)	2	28	−14	9.55
		L	FL (MeFG)	−2	22	−16	9.43
9	3,392	L	TL (ITG)	−50	−52	−8	9.54
		L	TL (MTF)	−54	−44	−10	9.50
10	2,672	L	FL (IFG)	−40	20	−16	9.51
		L	TL (STG)	−32	24	−24	9.47
11	2,376	L	PL (Precuneus)	−12	−56	44	9.51
12	1984	R	OL (FG)	23	−58	−8	9.40
13	1952	L	PoL	−22	−58	−12	9.55
14	1936	R	Sub-lobar	30	18	6	9.50
15	1928	R	PL (IPL)	46	−50	40	9.49
16	1920	R	OL (LG)	4	−86	0	9.47
17	1912	R	FL (MeFG)	2	−14	64	9.51
18	1904	L	FL (Prec)	−42	4	37	9.49
19	1896	R	FL (IFG)	39	20	−18	9.48
20	1880	R	FL (MFG)	42	2	38	9.50
21	1,608	R	PoL	46	−68	−30	9.47
22	1,600	R	FL (SFG)	30	54	−4	9.50
23	1,504	L	FL (SFG)	−10	42	48	9.53
24	1,120	L	FL (SFG)	−17	62	24	9.44
25	864	R	FL (SFG)	12	66	18	9.48

PL, parietal lobe; FL, frontal lobe; LL, limbic lobe; SFG, superior frontal gyrus; CG, cingulate gyrus; MFG, middle frontal gyrus; AC, anterior cingulate; MeFG, medial frontal gyrus; TL, temporal lobe; STG, superior temporal gyrus; PG, parahippocampal gyrus; Prec, precentral gyrus; IFG, inferior frontal gyrus; PoG, postcentral gyrus; SG, supramarginal gyrus; ITG, inferior temporal gyrus; MTG, middle temporal gyrus; OL, occipital lobe; PoL, posterior lobe; FG, fusiform gyrus; IPL, inferior parietal lobule; LG, lingual gyrus.

**Table 4:** Single meta-analysis of love–envy.

Cluster#	Volume mm^3^	Hemisphere	Brain region	Peak coordinates (MNI coordinate system)			ALE Value (×10^−4^)
				X	Y	Z	
1	21,440	L	Sub-lobar	−8	2	0	9.56
		L	Midbrain	0	−14	−8	9.56
		R	Midbrain	12	−12	−2	9.56
		L	Sub-lobar	−18	−8	−2	9.55
		L	Sub-lobar	−12	2	2	9.55
		L	Sub-lobar	−24	−10	−2	9.55
		L	Sub-lobar	−12	12	2	9.55
		R	FL (IFG)	24	8	−18	9.55
		L	Midbrain	−6	−18	−2	9.54
		R	Midbrain	8	−18	−2	9.54
		L	Midbrain	−6	−12	−4	9.54
		L	Midbrain	−8	−14	−10	9.54
		R	Sub-lobar	12	0	−2	9.54
		R	Sub-lobar	6	−2	6	9.52
		L	Sub-lobar	−32	−28	−2	9.52
		R	LL	22	2	−12	9.51
		L	Midbrain	−16	−24	−2	9.51
		L	Sub-lobar	−34	−26	−6	9.51
		L	Sub-lobar	−12	−6	10	9.51
		L	TL	−30	−20	−10	9.51
		L	Sub-lobar	−8	−12	8	9.50
		R	Sub-lobar	18	−4	−2	9.48
		L	Sub-lobar	−6	10	−2	9.48
		L	LL	−24	−20	−6	9.48
		L	Sub-lobar	−18	8	−2	9.48
		R	Sub-lobar	2	−4	−10	9.47
		L	Sub-lobar	−20	−24	−2	9.47
		L	Sub-lobar	−6	−30	2	9.47
		L	Midbrain	−8	−18	−10	9.47
		L	Sub-lobar	−4	−24	6	9.46
		R	Midbrain	8	−12	−8	9.43
		L	Sub-lobar	−8	−6	12	9.42
		R	Sub-lobar	2	−10	8	9.41
		L	Sub-lobar	−2	−4	−10	9.41
		L	Sub-lobar	−14	12	6	9.40
2	4,096	R	PoL	20	−66	−16	9.54
		R	PoL	12	−74	−8	9.53
		R	PoL	10	−64	−22	9.52
		R	PoL	18	−68	−12	9.49
		R	PoL	24	−86	−19	9.47
		R	PoL	16	−80	−16	9.47
3	3,520	L	LL (CG)	−2	36	28	9.54
		L	LL (AC)	−4	26	24	9.52
		L	LL (AC)	−2	20	20	9.51
		R	FL (MeFG)	8	42	26	9.49
		L	FL (MeFG)	−2	44	22	9.39
4	2,584	R	LL (AC)	4	36	6	9.51
		L	LL (AC)	−2	36	−4	9.47
		R	LL (AC)	2	32	12	9.45
		L	LL (AC)	−2	32	14	9.40
5	2,520	R	TL (SG)	64	−46	24	9.49
		R	TL (STG)	50	−46	16	9.48
		R	TL (STG)	62	−48	16	9.48
		R	TL (STG)	60	−58	22	9.43
6	2,312	R	Sub-lobar	18	12	−6	9.51
		R	Sub-lobar	14	12	8	9.50
		R	Sub-lobar	8	12	4	9.43
7	2,224	R	LL (PG)	28	−22	−14	9.55
		R	Sub-lobar	34	−26	−6	9.51
		R	LL (PG)	24	−20	−6	9.48
8	1872	L	TL (MTG)	−46	−76	28	9.53
		L	TL (MTG)	−52	−70	22	9.50
		L	TL (MTG)	−40	−66	22	9.48
9	1848	L	PL (IPL)	−50	−50	38	9.52
		L	PL (IPL)	−50	−42	34	9.51
		L	PL (IPL)	−60	−46	40	9.45
10	1,648	R	LL (CG)	6	−18	34	9.53
		L	LL (CG)	0	−16	38	9.53
11	1,464	L	FL (IFG)	−38	14	−10	9.50
		L	Sub-lobar	−30	12	−8	9.47
12	1,456	L	FL (IFG)	−38	28	−2	9.47
		L	FL (IFG)	−44	28	6	9.47
13	1,456	L	PL (Precuneus)	−8	−60	22	9.50
		L	LL (PoC)	−8	−52	16	9.48
14	1,456	R	FL (SFG)	6	44	44	9.50
		R	FL (MeFG)	12	38	38	9.46
15	1,448	L	FL (Prec)	−14	−30	64	9.45
		L	FL (MeFG)	−2	−26	62	9.38
16	1,440	R	PL (IPL)	50	−36	32	9.54
		R	PL (SG)	40	−38	32	9.49
17	1,416	L	FL (MeFG)	−8	50	10	9.48
		L	FL (MeFG)	−4	50	4	9.47
18	1,400	L	TL (MTG)	−56	−48	6	9.53
		L	TL (STG)	−60	−52	16	9.49
19	1,344	L	FL (SFG)	−2	24	52	9.55
		L	FL (SFG)	−6	20	60	9.51
20	1,280	R	LL (PoC)	6	−54	22	9.56
		R	LL (PoC)	8	−52	16	9.48
21	880	L	FL (MFG)	−44	8	46	9.53
		L	FL (MFG)	−48	6	46	9.53
22	752	R	AL	6	−38	−4	9.48
23	752	L	Sub-lobar	−34	3	2	9.47
24	752	L	Sub-lobar	−38	18	12	9.46
25	736	L	PoL	−24	−86	−19	9.47
26	736	L	OL (MTG)	−44	−76	12	9.44
27	736	L	PL (SG)	−32	−50	32	9.44
28	736	L	LL (CG)	−12	−42	36	9.50
29	736	L	PL (PoC)	−30	−26	40	9.44
30	728	R	Sub-lobar	12	2	16	9.50
31	720	L	LL (PG)	−38	−48	−6	9.48
32	720	L	OL	−20	−70	12	9.47
33	720	R	Sub-lobar	18	−12	12	9.48
34	720	R	Sub-lobar	36	−34	20	9.48
35	720	L	LL (CG)	−18	−8	36	9.53
36	712	L	Sub-lobar	−44	14	0	9.52
37	712	L	FL (Prec)	−42	4	10	9.48
38	712	R	Sub-lobar	26	18	10	9.52
39	704	R	FL (IFG)	42	26	4	9.52
40	704	L	Sub-lobar	−40	−6	44	9.51
41	696	L	FL (AG)	−44	−62	34	9.53
42	688	L	LL (CG)	−18	−34	44	9.55
43	656	R	FL (SFG)	8	58	28	9.43
44	560	R	FL (IFG)	54	28	2	9.52
45	536	L	FL (MeFG)	−6	64	12	9.55

FL, frontal lobe; LL, limbic lobe; IFG, inferior frontal gyrus; PG, parahippocampal gyrus; TL, temporal lobe; PoL, posterior lobe; CG, cingulate gyrus; AC, anterior cingulate; MeFG, medial frontal gyrus; MFG, middle frontal gyrus; SG, supramarginal gyrus; STG, superior temporal gyrus; PL, parietal lobe; MTG, middle temporal gyrus; IPL, inferior parietal lobule; AL, anterior lobe; Precuneus; PoC, posterior cingulate; SFG, superior frontal gyrus; Prec, precentral gyrus; OL, occipital lobe; PoG, postcentral gyrus; AG, angular gyrus.

### 3.3 Joint analysis results

**Figure 4:**
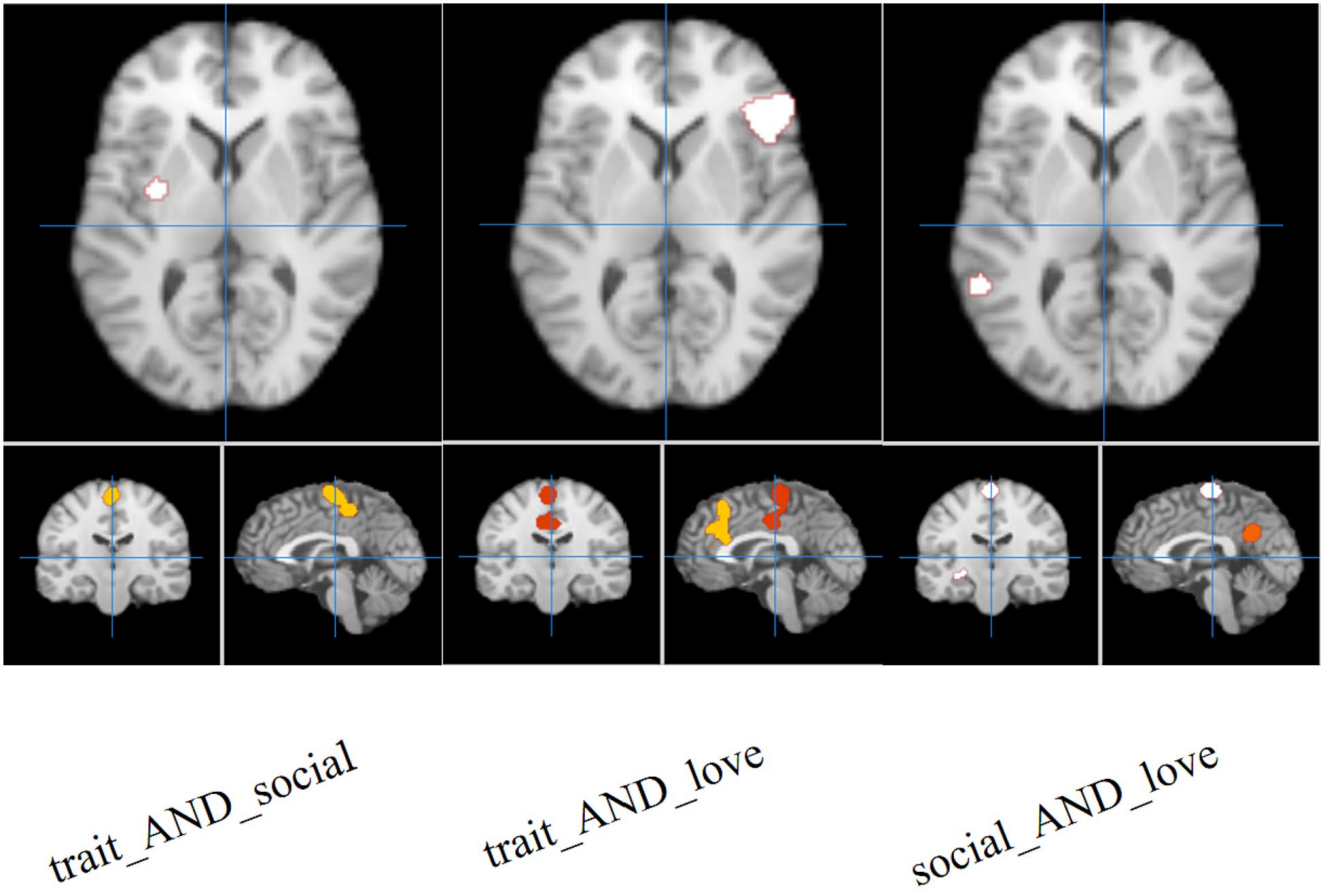
Joint analysis results. The intensity of activated brain regions in the figure gradually increases from red to white.

The results of the joint analysis showed that the fusion of trait (personality) envy and social comparison envy yielded a total of eight clusters, the fusion of trait (personality) envy and love–envy yielded a total of eight clusters, and social comparison envy and love–envy yielded a total of 10 clusters (Table 5). Specifically, trait (personality) envy and social comparison envy co-activate the following brain regions: right frontal sub-Gyral, right inferior parietal lobule, left inferior frontal gyrus, left precuneus, right paracentral lobule, left posterior lobule declive, right posterior lobule, left extra-nuclear lobule; trait (personality) envy and love–envy co-activate the following brain regions: left lobule extra the brain areas co-activated by trait envy and love–envy are: left extra-nuclear lobule, right sub-lobar lentiform nucleus, left paracentral lobule, left parietal supramarginal gyrus, left limbic cingulate gyrus, right inferior frontal gyrus, right parietal supramarginal gyrus, left middle frontal gyrus; brain areas co-activated by social comparison envy and love–envy are: left limbic anterior cingulate gyrus, right sub-lobar insula, right parietal supramarginal gyrus, left inferior frontal gyrus, cingulate gyrus, declive, middle frontal gyrus, temporal lobe sub-Gyral, and extra-nuclear lobule. The distribution of the activated brain areas jointly activated the brain between the two envy types is shown in Figure 4.

**Table 5:** Joint analysis of activation cluster results.

Conjunction	Volume mm^3^	Hemisphere	Brain region	Center coordinates			ALE Value (×10^−3^)
				X	Y	Z	
trait_AND_social	47,976	R	FL (sub-Gyral)	23.4	25.5	15.4	18
	12,160	R	PL (IPL)	48.9	−37.9	38.4	19
	8,248	L	FL (IFG)	−36	18.2	−13.9	18
	6,968	L	PL (Precuneus)	−15.9	−58.8	46.3	16
	6,896	R	FL (ParL)	2.7	−33.6	51.5	12
	3,912	L	PoL (Declive)	−16.9	−57.7	−12.9	15
	3,800	R	PoL	41.7	−65.4	−26.1	16
	1,592	L	Sub-lobar (EN)	−29.7	−6.6	−4.9	10
Trait_AND_love	33,216	L	Sub-lobar (EN)	−32.4	5.7	1.7	18
	18,832	R	Sub-lobar (LN)	24	−9.8	−6.1	17
	10,672	L	FL (ParL)	−6.3	−26.9	47.1	17
	8,664	L	PL (SG)	−44.3	−45	36.2	16
	8,624	L	LL (CG)	−4.1	25.3	30.4	16
	7,792	R	FL (IFG)	44.2	25	4.7	14
	6,928	R	PL (SG)	45.2	−36.7	34.7	17
	2,840	L	FL (MFG)	−11.9	48.1	17.2	13
Social_AND_love	23,680	L	LL (AC)	−1.8	36.6	13.6	19
	9,456	R	Sub-lobar (Insula)	36.9	19.8	4.9	18
	7,376	R	PL (SG)	51.7	−37.4	32.8	18
	6,864	L	FL (IFG)	−36.2	15.3	−12.4	17
	5,496	L	LL (CG)	−1.3	−57.3	26.1	16
	4,800	R	PoL (Declive)	16.1	−69.1	−8.9	14
	4,488	L	FL (MFG)	−42.4	3.3	41.4	16
	4,280	L	TL (sub-Gyral)	−48.2	−49.2	−4.9	14
	3,712	L	Sub-lobar (EN)	−27.1	−11.3	−9.2	13

FL, frontal lobe; PL, parietal lobe; IPL, inferior parietal lobule; IFG, inferior frontal gyrus; Precuneus; ParL, paracentral lobule; PoL, posterior lobe; EN, extra-nuclear; LN, lentiform nucleus; SG, supramarginal gyrus; LL, limbic lobe; CG, cingulate gyrus; IFG, inferior frontal gyrus; MFG, middle frontal gyrus; AC, anterior cingulate; TL, temporal lobe.

### 3.4 Contrasting analysis results

The results of the comparative analysis showed no activation clusters in the two-by-two comparative analysis of three envy types. To reduce the possibility of type II statistical errors, ALE comparative analysis was also validated using very loose thresholds (no correction threshold, *p* < 0.05, and a minimum activation cluster size of 100 mm^3^), and no significant differences were found.

## 4 Discussion

This study analyzed 13 existing functional magnetic resonance imaging studies of three types of envy – trait (personality) envy, social comparison envy, and love–envy – to observe the similarities and differences in the brain regions involved in the three types of envy processing and identify the neural mechanisms underlying the processing of the three types of envy.

### 4.1 Single-unit analysis: neural mechanisms of different types of envy

The single-unit analysis showed that 45 peak copula classes of trait (personality) envy yielded 20 clusters. The key brain regions that were activated included the inferior frontal gyrus, cingulate gyrus, middle frontal gyrus, lentiform nucleus, inferior parietal lobule, declive, and superior frontal gyrus.

Ochsner and Gross (2007) constructed a cognitive control model of emotion regulation with a bottom-up and top-down perspective. The brain neural network uses a bottom-up approach to encode the dynamic properties of stimuli, evaluate different types of emotions, and generate different types of emotional responses; it performs the evaluation of emotional stimuli and the control of emotional expression or experience in a top-down manner, regulating them, channeling them, and changing how emotional stimuli are evaluated.

Our account of how envy is generated is multi-leveled and bottom-up in its description of both the processes and the neural systems that give rise to emotional response. In the first step, a stimulus is perceived in its current situational context. The lentiform nucleus is the core region of the vertebrate neural circuit,” and its activity is enhanced when exposed to negative emotional stimuli (Deng et al., 2015). In the included literature, researchers used the Multidimensional Jealousy Scale (MJS) and the Dispositional envy scale (DES) to measure participants’ levels of trait envy. Example of statements include, “I feel envy every day,” and “Feelings of envy constantly torment me.” During the measurement, participants were asked to recall instances of envy. This led to activation of the lentiform nucleus. At the second stage, one attends to some of these stimuli or their attributes and appraises the significance of stimuli in terms of their relevance to one’s current goals, wants or needs. The cingulate gyrus is located in the “core limbic brain cluster,” a central structure responsible for integrating emotions, responding to emotional information and encoding information about emotionally salient events (Moraweta et al., 2017). Therefore, it is associated with the trait (personality) envy. When recalling instances of envy, the subjects emotionally encoded them. Finally, the third stage involves translating these appraisals into changes in experience, emotion-expressive behavior, and autonomic physiology.

With an understanding of how emotions are generated in the first place we can turn to an account of the process and neural systems involved in regulating them. Emotional regulation is top-down. The superior frontal gyrus and inferior parietal lobule are thought to be closely related to cognitive control and emotion regulation (Sheline et al., 2010; Thomas and Joseph, 2016). The generation of irrational envy accompanies the activation of both brain regions, and the levels of cognitive control and emotion regulation ability of different individuals affect their traits (personality). Studies have found that the middle and inferior frontal gyrus are crucial for regulating negative emotions through cognitive reappraisal strategies and expression inhibition (Buhle et al., 2014; Nimarko et al., 2019). The core operation of expression inhibition is the individual’s effort to inhibit emotion-related facial expressions when the stimulus has successfully evoked emotional expressions and physiological responses, such as breathing and heartbeat, to prevent emotions from being expressed when incentives have successfully produced them. The declive, as part of the cerebellar functional area, is involved in regulating muscle tone and coordinating the accuracy of casual movements (Dong et al., 2010) and may be related to the expression inhibition activity of facial expressions associated with trait (personality) envy.

**Figure 5:**
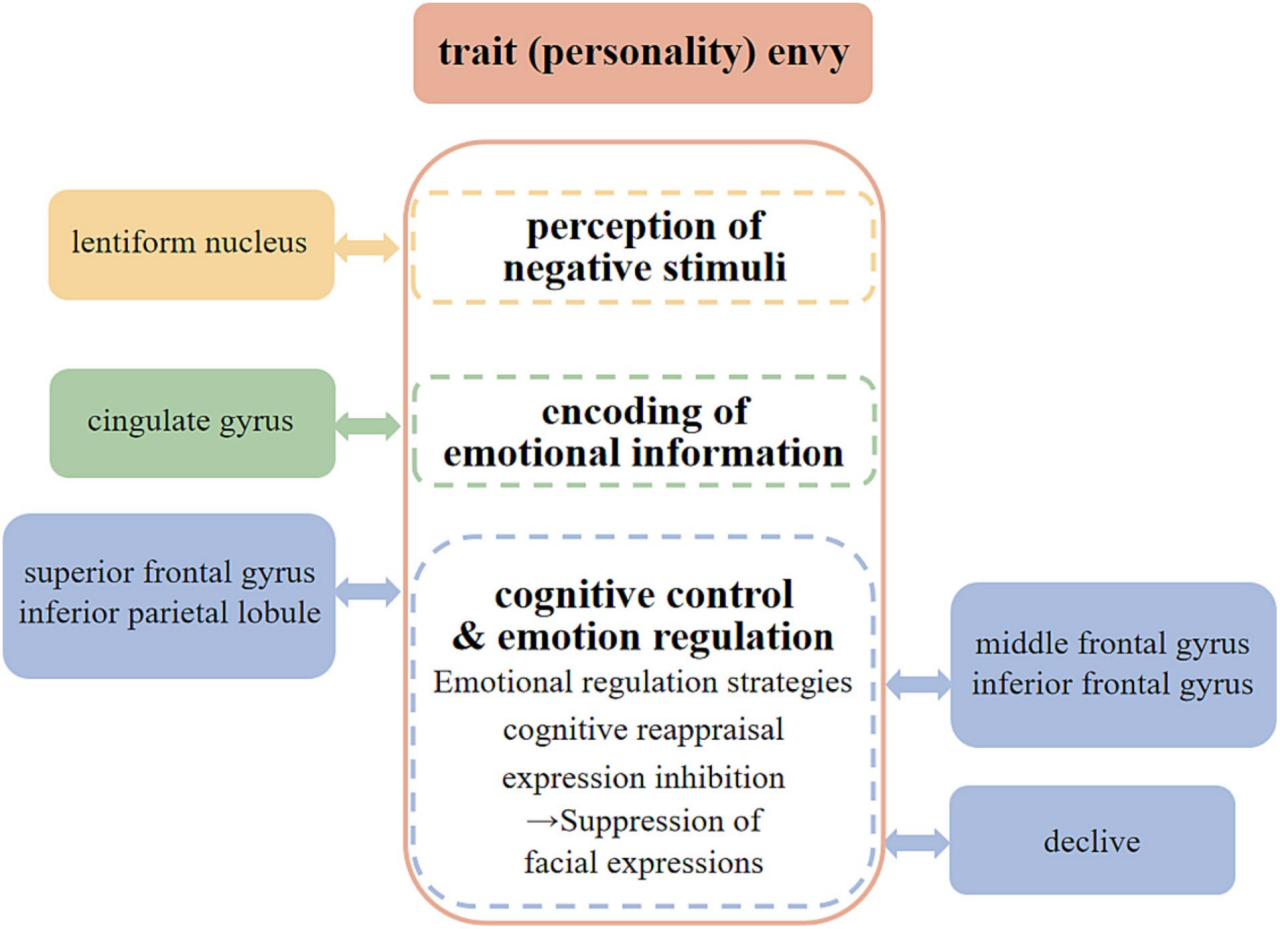
Neural mechanisms of the trait (personality) envy.

In summary, we can classify the neural mechanisms of trait envy into three levels: “perception of negative stimuli,” “encoding of emotional information,” and “cognitive control and emotion regulation” (see Figure 5).

The single-unit analysis showed that the social comparison envy of the 44 peak copolymer classes yielded 25 clusters. The key brain regions primarily activated were the middle frontal gyrus, inferior frontal gyrus, medial frontal gyrus, precuneus, inferior parietal lobule, precentral gyrus, superior temporal gyrus, declive, and anterior cingulate gyrus.

We can find that the model of the neural mechanisms of social comparison envy remains consistent with the cognitive control model of emotion regulation (Ochsner and Gross, 2007). Regarding the process through which envy generates, in the first step, a stimulus is perceived in its current situational context. The generation of social comparative envy is closely linked to the social comparative context. Previous research has shown that the precuneus acquires information and experiences from a first-person perspective in highly integrated tasks while participating in contextual memory extraction, self-reference, and social cognition (Cavanna and Trimble, 2006; Buckner and Carroll, 2007). Contextual memory extraction is associated with the story-context approach for inducing social comparison envy. In contrast, self-reference and social cognition fit the defining characteristics of social comparison envy based on the upward social comparison that triggers envy. The medial frontal gyrus includes the medial prefrontal cortical and orbitofrontal regions and is associated with cognitive functions such as purposeful decision-making, and reward and punishment reflexes (Zhu, 2002). The functional magnetic resonance imaging study included in this study used the money gain/loss game to induce social comparison envy. The experimental paradigm begins with cognitive functions related to purposeful decision-making and the reward or punishment reflex. At the second stage, one attends to some of these stimuli or their attributes and appraises the significance of stimuli in terms of their relevance to one’s current goals, wants or needs. The activation of the precentral gyrus is associated with oxytocin secretion (Chen, 2017), which influences individual emotion recognition. Olff et al. (2013) found that oxytocin enhances individual sensitivity to salience cues from the (social) environment (Bartz et al., 2011) or interpersonal (Ellenbogen et al., 2012), with the effect when salience cues are interpreted as “insecure” or “negative,” oxytocin may inhibit the recognition of negative emotions from promoting socially adaptive behavior. Salient cues in situations that can provoke social comparison envy are insecure and negative for the individual. It appears that the generation of individual social comparison envy accompanies the activation of the precentral gyrus. Finally, the third stage involves translating these appraisals into changes in experience, emotion-expressive behavior, and autonomic physiology.

Regarding the process through which envy regulation, similar to the trait (personality) envy, envy regulation is top-down. Social comparison envy activates the inferior parietal lobule, which is closely associated with cognitive control and emotional regulation. The superior temporal gyrus plays a very important role in emotion regulation and social cognitive processing (Song et al., 2019). The medial frontal gyrus is also associated with emotion regulation (Zhu, 2002). The activated anterior cingulate gyrus can integrate afferent information from different sources and regulates cognitive and emotional functions (Bush et al., 2000). The middle and inferior frontal gyri exert cognitive reappraisal and expression-suppression strategies in emotion regulation. The expression-suppression strategy triggers the declive to engage in expression-suppression activities of facial expressions associated with social comparison envy.

**Figure 6:**
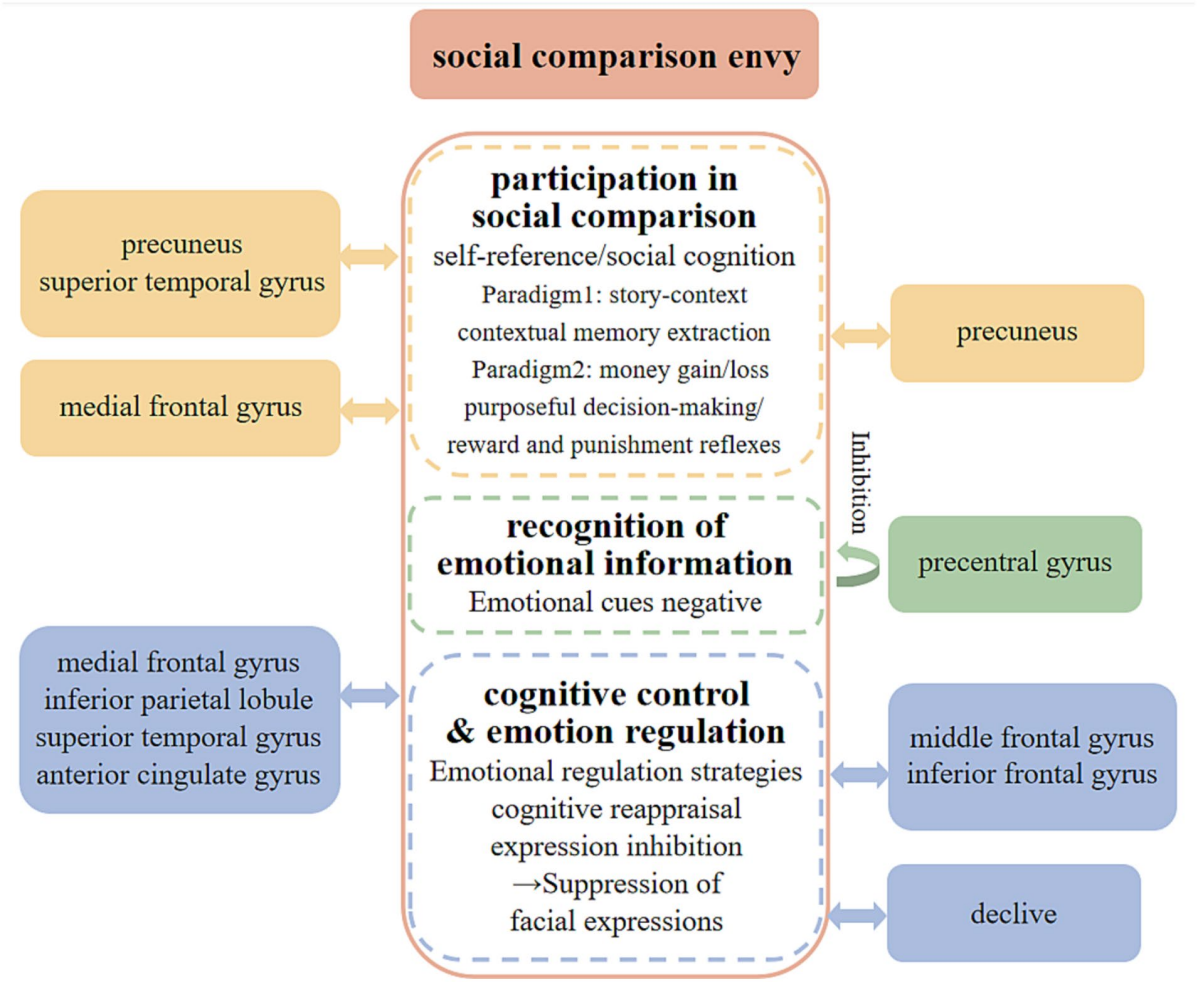
Neural mechanisms of social comparison envy.

In summary, we can classify the neural mechanisms of social comparison envy into three levels: “participation in social comparison,” “recognition of emotional information,” and “cognitive control and emotion regulation” (see Figure 6).

The single-unit analysis showed that 121 peak co-localization classes of love–envy yielded 45 clusters. The critical brain regions mainly activated include the inferior frontal gyrus, superior frontal gyrus, cingulate gyrus, insula, claustrum, medial frontal gyrus, inferior parietal lobule, caudate, and posterior cingulate gyrus.

We can find that the model of the neural mechanisms of social comparison envy remains consistent with the cognitive control model of emotion regulation (Ochsner and Gross, 2007). Regarding the process through which envy generates, in the first step, a stimulus is perceived in its current situational context. The generation of love–envy is closely linked to context related to sexual infidelity and emotional infidelity. The claustrum plays a vital role in human sexual arousal and problems (Redouté et al., 2000), which fit the defining characteristics of love–envy based on sexual partnerships. The present study found that love–envy activated the caudate. The caudate involves reward mechanisms, emotional processing, and motivation (Villablanca, 2010). Neuroimaging studies of romantic love have found significantly more robust functional connectivity in the reward-motivation network (caudate) in relationship groups than in single groups (Song et al., 2015), suggesting that the triggering of love–envy is associated with the activation of the reward-motivation network by romantic love. The activation of the medial frontal gyrus is also associated with cognitive functions such as reward and punishment responses (Zhu, 2002). At the second stage, one attends to some of these stimuli or their attributes and appraises the significance of stimuli in terms of their relevance to one’s current goals, wants or needs. The posterior cingulate gyrus is an assessment area (Bush et al., 2000) that integrates visual cognition in the visual cortex and emotional processes in the anterior cingulate gyrus in response to dynamic events (Lim et al., 2004; Enatsu et al., 2014; Morawetz et al., 2017). The love–envy brain imaging study selected for this study used an infidelity contextual utterance task. Peak visual cortical coordinates unrelated to envy and activated only by the evoked task were found with peak activation (removed). Similarly, posterior cingulate activation in visual-cortical visual-cognitive integration is task-related. Love–envy is induced by infidelity (sexual or emotional affairs) and dynamic events that activate the posterior cingulate gyrus. Therefore, love–envy is associated with the posterior cingulate gyrus. Finally, the third stage involves translating these appraisals into changes in experience, emotion-expressive behavior, and autonomic physiology. The insula is thought to represent a viscerotopic map of ascending viscerosensory inputs from the body (Mufson and Mesulam, 1982) and has been implicated in negative affective experience in general (Craig, 2009). There appears to be implicated in negative affective in the insula with posterior regions associated with primary representations of sensations from the body and anterior regions associated interoceptive awareness of the body and in motivational and affective states, like envy, that have a strong visceral component (Craig, 2009).

Regarding the process through which envy regulation, similar to the trait (personality) envy and social comparison envy, envy regulation is top-down. The insula is involved in various tasks related to emotional regulation and cognitive control (Cauda et al., 2012). Similar to the first two types of envy, love–envy activates the superior frontal gyrus, the medial frontal gyrus, and inferior parietal lobule, which are closely related to cognitive control and emotion regulation. The inferior frontal gyri exert cognitive reappraisal and expression-suppression strategies in emotion regulation.

In summary, we can divide the neural mechanism of love–envy into three levels: “love–envy elicitation,” “evaluation of emotional information,” and “cognitive control and emotion regulation” (Figure 6).

In terms of the mechanisms that generate the three types of envy, each of them is unique when it comes to the perception of stimuli in a context. As we can see from the neural mechanism models of social comparison envy and love envy, compared to the trait (personality) envy, social comparison envy and love–envy as two types of state emotions generated by specific experimental tasks, although using different types of experimental tasks, i.e., inducing social comparison envy using the story context method and the money gain/loss game, and inducing love–envy using the infidelity contextual utterance task and the contextual imagery tasks, but they all fit their respective definitions. Among them, social comparison envy was associated with brain areas of self-reference and social cognition, and love–envy was associated with brain areas of sex, reward, and motivation.

In terms of the emotion regulation mechanisms of envy, the three types of envy share very similar neural mechanisms. The cognitive control model of emotion regulation suggests that emotion regulation arises during the process of emotion onset and that different emotion regulation occurs at different stages of emotion onset (Gross and James, 1998; Gross and Thompson, 2007). Among them, cognitive changes are formed before the formation of emotional response tendencies, which are prior-focused emotion regulation and exhibit cognitive reappraisal emotion regulation strategies; response adjustments are made after the formation of emotional response tendencies, which are response-focused emotion regulation and exhibit expression inhibition of emotion regulation strategies. A single-unit analysis found that all three envy types induced brain regions associated with cognitive reappraisal and expression-inhibiting emotion regulation strategies, including the middle frontal gyrus, the inferior frontal gyrus, and the slope of the cerebellum. It is evident that when people develop envy, they use cognitive reappraisal to understand the adverse emotional event more positively or to rationalize the emotional event. Expressive inhibition is also used to mobilize self-control and to initiate self-control processes to inhibit one’s emotional behavior.

### 4.2 Joint analysis: the relationship of neural mechanisms between different types of envy

Joint analysis showed that the fusion of trait (personality) and social comparison envy yielded eight clusters. The key brain regions mainly activated included the frontal sub-gyrus, inferior parietal lobule, inferior frontal gyrus, precuneus, paracentral lobule, declive, posterior lobule, and extra-nuclear lobule. The single-unit analysis shows that both envy types have sub-parietal lobules closely related to cognitive control and emotion regulation. Simultaneously, the inferior frontal gyrus influences mental reappraisal and expression inhibition in emotion regulation strategies. The expression suppression strategy triggers the involvement of the declive in the suppression of facial expressions associated with trait (personality) envy and social comparison envy. It was found that the precuneus, activated significantly after a joint analysis of the two envy types, was not activated considerably during a single meta-analysis of trait (personality) envy but was activated substantially during a single meta-analysis of social comparison envy. The precuneus is also involved in self-information processing related to the self (Northoff et al., 2006). The functional magnetic resonance imaging study of trait (personality) envy included in this study was measured by a scale with self-relevant items, such as “I feel jealous every day, and the feeling of envy torments me constantly” from the Dispositional Envy Scale. Noteworthy, trait (personality) envy may activate the precuneus lobe. However, in contrast to social comparison envy, which is based on the contextual characteristics of upward social comparison triggering envy, the precuneus activation of the trait (personality) envy originates only from the measurement modality and not from the type of envy itself. Therefore, in the single meta-analysis, precuneus activation was insignificant within the activated brain regions for trait (personality) envy compared with other brain regions. The joint analysis also identified significantly activated brain regions not found in either envy type in the single meta-analysis: frontal sub-gyrus, paracentral lobule, posterior lobe, and extra-nuclear lobule. Among them, the brain regions associated with emotion regulation and cognition are the frontal sub-Gyral and posterior lobes (lobules VI and VII; Phillips et al., 2008; Mu et al., 2022), the paracentral lobule is involved in self-related information processing (Yang, 2016), and the lateral lobule nucleus cluster includes the lateral amygdala, basal amygdala, and parabasal amygdala, which are considered the main structures that provide information for emotion perception (Han, 2016). They were all associated with trait (personality) envy and social comparison envy; however, their role as a single envy type was insignificant.

On the one hand, it may be that other brain regions with the same function (e.g., sub-parietal lobule, precuneus, and lentiform nucleus) are relatively overshadowed by the more significant effect sizes. On the other hand, the paracentral envy function of processing information related to the self is not substantial in the trait (personality) envy, as it only originates from the measurement modality and not from the envy type itself. The function of the amygdala in providing emotion perception information in social comparison envy was more often completed when brain regions associated with social comparison were involved in the evoked paradigm and thus was not significant.

The joint analysis showed that trait (personality) envy and love–envy fusion yielded eight clusters. Critical brain regions that were mainly activated included the extra-nuclear lobule, lentiform nucleus, paracentral lobule, cingulate gyrus, inferior frontal gyrus, supramarginal gyrus, and middle frontal gyrus. The single meta-analysis clearly showed that both envy types have cingulate gyri that carry out emotional information responses and encode information about emotionally salient events, which influences the cognitive reappraisal strategy, and the inferior frontal gyrus, which expresses the inhibition strategy in the emotion regulation strategy. It was found that the lentiform nucleus and middle frontal gyrus, activated significantly when both envy types were analyzed jointly, were not markedly activated in the love–envy single meta-analysis but were activated mainly in the trait (personality) envy single meta-analysis. The role of the lentiform nucleus in the perception of negative emotions in love–envy was more often completed when brain regions associated with romantic love were involved in the evoked paradigm and, therefore, was not significant. The middle frontal gyrus has the same function as the inferior frontal gyrus. The inferior frontal gyrus’s effect on love–envy may be more meaningful and relatively overshadow the impact of the middle frontal gyrus; therefore, it is not essential. The joint analysis also identified significantly activated brain regions not found in either envy type during the single meta-analysis, namely the paracentral lobule, extra-nuclear lobule, and limbic supramarginal gyrus. The paracentral lobule is involved in information processing related to the self; the amygdala in the lateral lobule cluster provides information on emotion perception (Han, 2016), and the supramarginal gyrus, a component of the inferior parietal lobule, is closely related to cognitive control and emotion regulation in common with it (Caspers et al., 2006). They are associated with both trait (personality) envy and love–envy; however, their role as a single envy type is insignificant.

On the one hand, other brain regions with the same function (e.g., the inferior parietal lobule) may be relatively masked by the more significant effect. On the other hand, the paracentral lobule’s function of processing information related to the self in the trait (personality) envy originates only from how it is measured and not from the envy type itself and is therefore not significant. The function of the amygdala in providing information about emotional perception is often accomplished in love–envy when the brain regions associated with romantic love are involved in the evoked paradigm and are therefore not significant.

The joint analysis results showed that 10 clusters were obtained for social comparison envy and love–envy fusion. The key centrally activated brain regions included the anterior cingulate gyrus, insula, supramarginal gyrus, inferior frontal gyrus, cingulate gyrus, declive, middle frontal gyrus, temporal lobe sub-Gyral, and extra-nuclear lobule. The single meta-analysis results clearly showed that both envy types influenced the cognitive reappraisal strategy of emotion regulation and expression inhibition strategy of the inferior frontal gyrus. It was found that the anterior cingulate gyrus, middle frontal gyrus, and declive, significantly activated after the joint analysis of both envy types, were not particularly activated during the single meta-analysis of love–envy. Nonetheless, they were activated considerably during the single meta-analysis of social comparison envy. The anterior cingulate gyrus is closely associated with cognitive control (Meldrum et al., 2018). In love–envy, the insula, superior frontal gyrus, medial frontal gyrus, and inferior parietal lobule have similar functions. The effect sizes of these brain regions may be more significant and mask the role of the anterior cingulate gyrus; therefore, they are not necessary. The middle frontal gyrus has the same function as the inferior frontal gyrus. However, the inferior frontal gyrus’s effect is possibly more significant in love–envy and overshadows the impact of the middle frontal gyrus; thus, it is not substantial.

The declive is associated with the “inhibitory” emotion regulation strategy. It has been found that expressing love–envy is more acceptable than expressing social comparison envy (Zhang et al., 2011). Individuals are less likely to use the “inhibitory” strategy for emotion regulation after love–envy is induced. The possibility of using the “expression inhibition” strategy for emotion regulation after the induction of love–envy was low and, therefore, insignificant. Simultaneously, the cingulate gyrus and insula, activated significantly after joint analysis of the two envy types, did not start considerably during the social comparison envy single meta-analysis but started especially during the love–envy single meta-analysis. The cingulate gyrus, associated with emotional information responses and information encoding emotionally salient events, was similarly activated during the social comparison envy evocation. However, in the single meta-analysis, it was found that the precentral gyrus inhibited the cingulate gyrus from identifying negative emotions more significantly in social comparison envy, which may have caused the cingulate gyrus to be insignificant in the single meta-analysis of social comparison envy and is consistent with the preference of social comparison envy for “inhibitory” emotion regulation strategies. The joint analysis also identified significantly activated brain regions that were not found in either type of envy in a single meta-analysis, namely the extra-nuclear lobule, supramarginal gyrus, and temporal lobe sub-gyrus, where the amygdala in the lateral lobule nucleus provides emotional perception information (Han, 2016) and the supramarginal gyrus is closely related to cognitive control and emotion regulation (Caspers et al., 2006) and the temporal lobe is involved in cognitive information processing, situational memory encoding, and extraction processes (Chong et al., 2020). On the one hand, it may be that other brain regions with similar functions (e.g., inferior parietal lobule and superior temporal gyrus) were obscured by a more significant effect size. On the other hand, possibly, the role of the amygdala in providing emotional perception information was already completed when brain regions associated with social comparison in social comparison envy and romantic love–envy were activated in the evoked paradigm, which may explain why the amygdala was not found to be not significant in these contexts.

### 4.3 Contrasting analysis: the relationship of neural mechanisms between different types of envy

The results of the comparative analysis showed no activation clusters in the comparisons of the three types of envy. Possible reasons for this are as follows: first, the inclusion criteria for the ALE study literature are somewhat subjective. Second, according to the inclusion criteria, fewer papers met the inclusion criteria in this study, and the statistical validity may be weak, resulting in insignificant differences among the three envy types.

### 4.4 Limitations and outlook

There are some limitations to our work. First, limited by the number of existing studies, we did not find significant differences between the three types of envy. With the abundance of related studies, it is possible to clarify the characteristics of the three envy types using only the corresponding neural processing mechanisms in the future. Second, there is the problem of publication bias that should be addressed. Coordinate- based neuroimaging meta-analyses test for spatial convergence of effects across experiments with the null-hypothesis of random spatial convergence (Rottschy et al., 2012). Thus a limitation of most coordinate-based algorithms is that they are insensitive to non-significant results and publication bias may go unnoticed. Most of the articles related to ALE meta-analysis have not been tested for publication bias. This may be due to the inability to perform traditional publication bias tests using coordinates. In the study, we uses the main effect size of each literature to judge the problem of publication bias. There may be limitations to this approach. Third, unfortunately, there is currently no option for correlation analysis in GingerALE. Also, the small number of included literature is the impossibility to not only calculate one main meta-analysis, but rather also sub-analyses which may focus on more specialized processes (e.g., different paradigm classes) or groups (e.g., different samples). Due to this reason, we cannot control variables sufficiently to minimize the influence of potential factors on neuropsychological mechanisms. Finally, in addition to the three common types of envy in this study, researchers have focused on other types of envy, such as good-intentioned envy, as proposed by the dual structural theory of envy (Crusius et al., 2020). Regarding motivational and behavioral tendencies, when confronted with the envied person’s superiority, individuals with good-intent envy will generate positive motivation that drives them to improve themselves through efforts; experienced envy or attributed envy based on emotional self-bias theory, among others. However, we did not include these newer types of envy in our literature inclusion because there has not been sufficient correlational research on fMRI to support the meta-analysis.

In the future, there is much work should to be done. First, our meta-analysis focused on the neural mechanisms of envy in healthy participants only. However, research has also been conducted on the neural mechanisms of envy in populations with autism, juvenile delinquents, and others (Daniel et al., 2021; Sol et al., 2023). Therefore, an important direction for future research is the translation of basic research on the generation and regulation of envy to understanding the full range of normal to abnormal differences in emotional generation and regulatory ability of envy. This is critical both for understanding the mechanisms underlying this variability and for testing the boundaries of basic models of envy generative and regulatory mechanisms. Second, one domain in which this will prove important is understanding how our envy changes as we grow from childhood through adolescence into adulthood and old age. The age of the participants in the literature included in this paper ranged from 17 to 27 years old. On the one hand, there is growing evidence that childhood and adolescence are critical times for the development of the envy regulatory abilities needed to adaptively regulate affective impulses and the deleterious offensive behavior they can promote (McRae et al., 2012). One the other hand, while physical health and cognitive abilities tend to decline with age (Grady, 2008), older adults report more emotional stability and a greater ratio of positive to negative experiences in their daily life, with the extent of positive emotion predicting longevity (Carstensen and Mikels, 2005). One conundrum to resolve here will be the apparent dependence of emotion regulation on the same kinds of prefrontal control systems that decline with age. This raises the question of how regulatory abilities improve as the underlying neural machinery declines (Ochsner et al., 2012). Early results suggest that it may depend on the strategies older adults deploy, with spared or greater regulatory ability shown for strategies and tactics that fit with long-term goals and have become habitual (Ochsner and Gross, 2008). Third, an important goal for future research will be to understand how potential dysfunction in the mechanisms of envy generation and regulation may underlie various forms of psychiatric and substance use disorders. This future direction is being pursued in studies across various disorders, ranging from delusional symptoms to depression and autism. These studies can be useful in two ways. First they may show disorder-specific patterns of altered function in control and affect systems. Second, imaging methods for studying emotion regulation may be used before and after treatment regimes as predictors of and markers of improvement.

## Data availability statement

The datasets presented in this study can be found in online repositories. The names of the repository/repositories and accession number(s) can be found in the article/supplementary material.

## Author contributions

SD: Conceptualization, Data curation, Formal analysis, Investigation, Methodology, Project administration, Software, Validation, Visualization, Writing – original draft, Writing – review & editing. QL: Conceptualization, Data curation, Funding acquisition, Investigation, Methodology, Project administration, Resources, Supervision, Validation, Visualization, Writing – original draft, Writing – review & editing. HC: Conceptualization, Formal analysis, Methodology, Project administration, Resources, Software, Supervision, Validation, Visualization, Writing – review & editing. WZ: Conceptualization, Funding acquisition, Resources, Supervision, Validation, Visualization, Writing – review & editing.

## Footnotes

^1^ http://brainmap.org/ale/

^2^ http://ric.Uthscsa.edu/mango/
